# Exploring the antimicrobial properties of dark-operating ceramic-based nanocomposite materials for the disinfection of indoor air

**DOI:** 10.1371/journal.pone.0224114

**Published:** 2019-10-23

**Authors:** Aliénor Dutheil de la Rochère, Alexeï Evstratov, Sandrine Bayle, Lionel Sabourin, Arnaud Frering, José-Marie Lopez-Cuesta

**Affiliations:** 1 Centre des Matériaux des Mines d’Alès, IMT-Mines Alès, Alès, France; 2 Laboratoire de Génie de l’Environnement Industriel, IMT-Mines-Alès, Alès, France; Institute of Materials Science, GERMANY

## Abstract

As people spend more and more time inside, the quality of indoor air becomes crucial matter. This study explores the germicidal potential of two dark-operating germicidal composite materials designed to be applied for the indoor air disinfection under flow conditions. The first material, MnO_2_/AlPO_4_/γ-Al_2_O_3_ beads, is a donor-acceptor interactive composite capable of creating hydroxyl radicals HO∙. The second one is a ZnO/γ-Al_2_O_3_ material with intercropped hexagons on its surface. To determine the antimicrobial efficiency of these materials in life-like conditions, a pilot device was constructed that allows the test of the materials in dynamic conditions and agar diffusion inhibitory tests were also conducted. The results of the tests showed that the MnO_2_/AlPO_4_/γ-Al_2_O_3_ material has a germicidal effect in static conditions whereas ZnO/γ-Al_2_O_3_ does not. In dynamic conditions, the oxidizing MnO_2_/AlPO_4_/γ-Al_2_O_3_ material is the most efficient when using low air speed whereas the ZnO/γ-Al_2_O_3_ one becomes more efficient than the other materials when increasing the air linear speed. This ZnO/γ-Al_2_O_3_ dark-operating germicidal material manifests the ability to proceed the mechanical destruction of bacterial cells. Actually, the antimicrobial efficiency of materials in dynamic conditions varies regarding the air speed through the materials and that static tests are not representative of the behavior of the material for air disinfection. Depending on the conditions, the best strategy to inactivate microorganisms changes and abrasive structures are a field that needs further exploration as they are in most of the conditions tested the best way to quickly decrease the number of microorganisms.

## Introduction

In developed countries, people spend more than 85% of their time in enclosed areas [1, 2]. In this context, the indoor air conditioning (climatic, chemical, and antimicrobial) is currently one of the strategic priorities in the domain of collective hygiene and healthcare.

Among modern technologies applied for the indoor air antimicrobial conditioning the greatest attention is currently drown to the photocatalytic air recycling procedures [3, 4]. Since Matsunaga *et al*. started to explore titanium dioxide TiO_2_ for bacteria inactivation [5], photocatalysts have been placed on the front position. However, all photocatalysts are energy-dependent materials; they need to be activated by external energy inputs. For voluminous confined spaces, the energy costs of recycling photocatalytic long-duration processes become significant. Moreover, photocatalytic reactions taking place at the surface of an active material need time to be achieved [6, 7], and in air conditioning procedures the contact time between the disinfecting agent and the air is a critical factor.

The possibilities of application of non-photocatalytic dark-operating active materials for the environmental media germicidal conditioning have already been discussed [8, 9]. These species occurring, in the majority of cases, as metal or metal oxide-based nanomaterials, including free nanoparticles, are declared to be energetically independent: no external energetic assistance is needed for their functioning. The oxidative stress provided by reactive oxygen species (ROS) formed in contact of metal or metal oxide-based nanomaterials and nanoparticles surfaces with humid media is the most widely probed contributory factor to the germicidal ability of the materials under consideration. The second mechanism, which can cause cell lysis by mechanical destruction of the cell membrane, is available for certain fibrous and tube-like shaped species [10, 11]. To this day, in the context of environmental application, germicidal materials have been applied predominantly in aqueous environment and often in static operating conditions [8, 12]. These conditions allow a high contact time between the material and the biological contaminant. Yet results depend on the test method [13] and this type of tests is by design unable to evaluate the efficiency of abrasive materials. For instance, dynamic tests have been led on silver-enhanced composites [14, 15] to approach life-like conditions and short contact time. Recently, real-time monitoring of microorganisms in air has become a new form of efficiency evaluation for air treatment and conditioning systems [16, 17].

The aim of this study was to evaluate the germicidal potential of two new dark-operating germicidal materials (DOGM), a MnO_2_-based interactive reactive oxygen species generator (ROS-DOGM) and a ZnO-based desert-rose-shaped cellular destructor (Mecha-DOGM), which were designed and synthesized for the purpose of this study. Manganese dioxide was chosen because of its ability to generate hydroxyl radicals without any energetic assistance [18] whereas zinc oxide has been selected for its crystalline structure which allows the creation of a wide diversity of structures [19, 20]. To avoid any powder release in the treated air, the active compounds, MnO_2_ and ZnO, were synthesized on the surface of macroscopic alumina beads. The used methods of synthesis allowed a chemical attachment of the surface oxide components to the host support. This study describes the synthesis of these composite materials as well as the evaluation of their germicidal properties using two different test methods: the agar diffusion inhibitory test and a dynamic test method developed to approach life-like conditions.

## Materials and methods

### Preparation of a ZnO-coated composite material

3*10^−3^ meters of diameter γ-Al_2_O_3_ beads were used as solid support. In order to synthesized ZnO nanostructures on the surfaces of these beads, 0.5 liter of an equimolar 0.1M solution of methenamine C_6_H_12_N_4_ (ChemLab) and zinc nitrate Zn(NO_3_)_2_⋅6H_2_O (ChemLab) was heated in contact with 300g of γ-alumina beads at 368 K for 10 hours. This method of synthesis was inspired by Vayssieres et al., 2001 [21]. After the heating step, the treated beads were rinsed thoroughly and were matured in air at room temperature for 24 hours. Then the beads were dried for 12 hours at 393 K and finally they were calcined for 4 hours at 823 K.

### Preparation of a MnO_2_/AlPO_4_/γ-Al_2_O_3_ composite material

3*10^−3^ meters of diameter γ-Al_2_O_3_ beads were also used as a solid support. Firstly, a layer of aluminum phosphate AlPO_4_ was synthetized by putting the alumina beads in a diluted solution of phosphoric acid (10%, Carlo Erba) for five minutes [22]. For the MnO_2_ coating, a 5 wt% manganese sulfate MnSO_4_^∙^H_2_O (Riedel-de Haën AG) solution was applied by incipient wetness impregnation. 0.288 liter of impregnating solution was used for 300g of alumina beads. After the impregnation step, the treated beads were subjected to maturation in air at room temperature for 24 hours, were dried for 6 hours at 393 K and were calcined for 4 hours at 823 K.

### Physico-chemical characterization of the elaborated materials

The elaborated composite materials were characterized using scanning electron microscope (SEM) Quanta 200 SEM / FEG (Field Emission Gun) with back-scattered electrons (BSE) detector. An energy-dispersive X-ray spectroscopy analysis (EDX) was also conducted to assess the chemical composition of the surface of the synthesize composite materials. The surface areas of the samples were measured using the Tristar II PLUS (Micrometrics) with N_2_ adsorption at 77K.

### Pilot installation for the test of the elaborated samples as germicidal agents for indoor air conditioning

**Fig 1:**
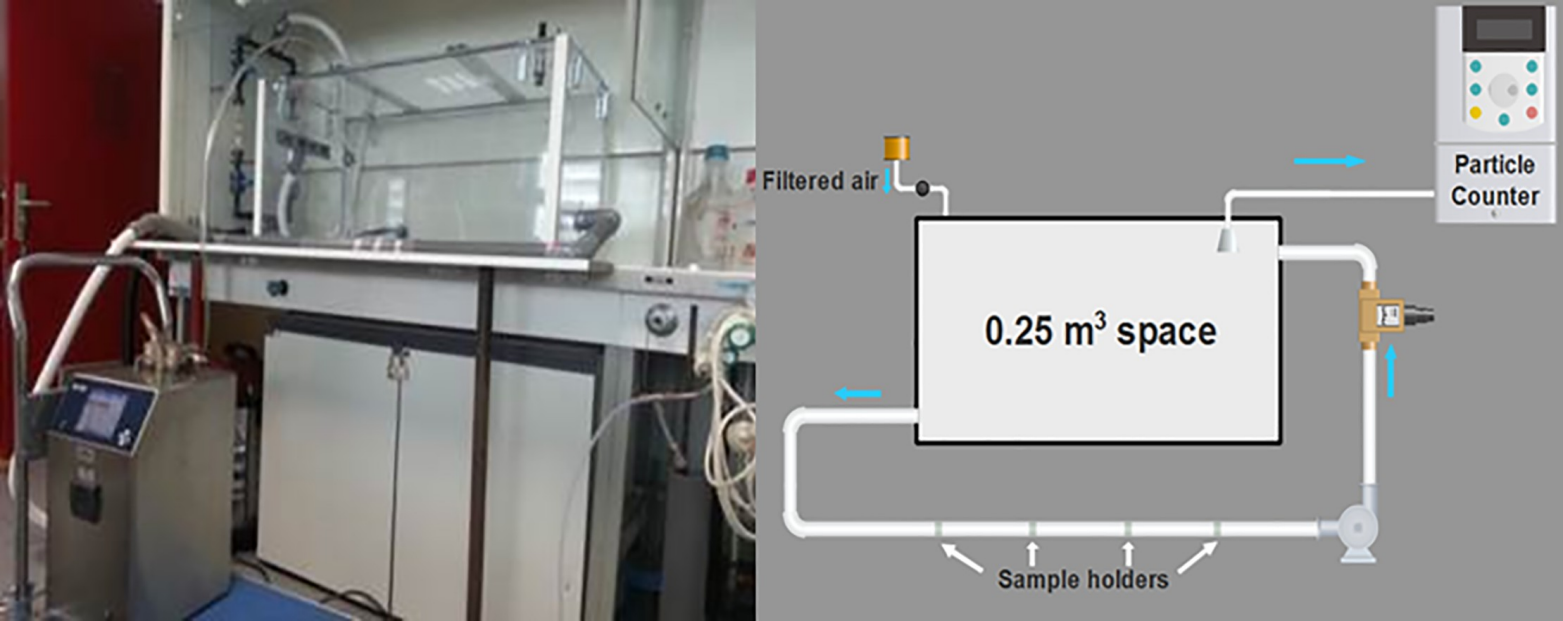
Photo and scheme of the pilot installation. The flow sensors are represented by a single flow rate meter (0.5 microns filtered air can enter the pilot unit to avoid a decrease of pressure).

The test device was designed to simulate an enclosed space with an external air renewal system. It was made up of four parts: a model space, sample holders, an air control unit and a particle counter. This model space had the following dimensions: 1.0 meter long, 0.5 meter wide and 0.5 m in height (total volume– 0.25 m^3^). It has been made in transparent 4_*_10^−3^ m thick polymethylmethacrylate. Sample holders were made using polyvinyl chloride (PVC) cylinders (45_*_10^-3^m of inner diameter, 1.2_*_10^-2^m of height) and fiberglass grids. A photo and a scheme of the pilot installation are shown in Fig 1.

**Fig 2:**
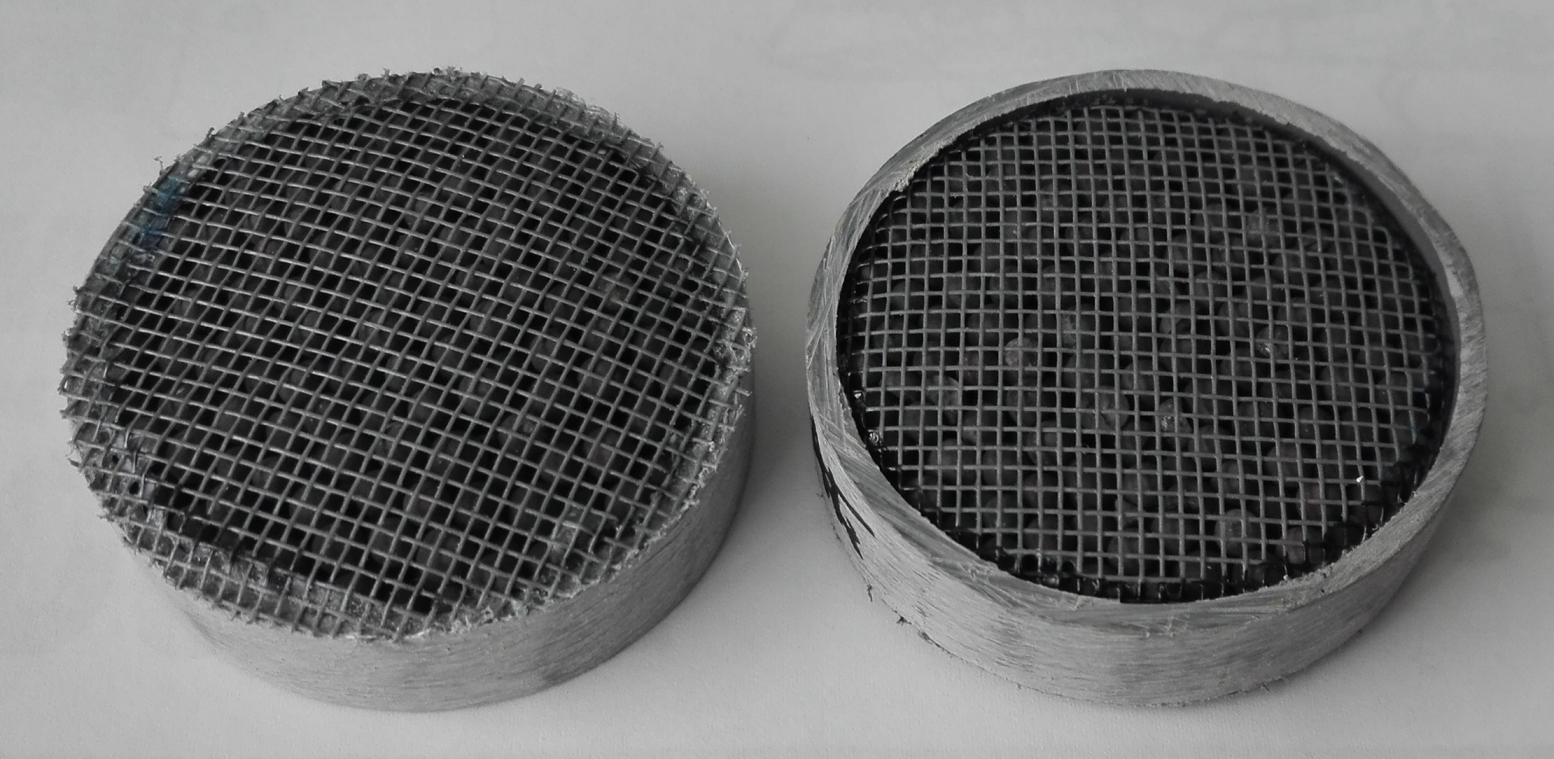
Front and back views of the sample holders.

The experiments were carried out using four sample holders (PVC cylinders) filled with active material. Each cylinder contained approximately 500 beads. 5.0_*_10^−2^ m of inner diameter. PVC tubes were used to maintain the sample holders in the desired configuration (Fig 2).

To increase the linear air speed while keeping the same air flow rate, PVC tubes with an inner diameter equal to 1.9_*_10^−2^ m were also applied. The air pump was an Einhell TH-VC 1930 SA without filter linked to two power transformers in series. As the Einhell TH-VC 1930 SA has a synchronous motor, the use of the transformers allowed variations of the flow rate in the test device. In addition, four Trogamid® variable-area flowmeters connected in parallel were used.

The particle counter BioTrak 9510-BD collected its samples from the 0.25 m^3^ space to which it was linked.

Several flow rates were applied: [1.0–1.25] m^3^/h, [2.0–2.5] m^3^/h and [4.0–4.25] m^3^/h. As it was pointed out before, PVC tubes of different widths were also used: 4.5*10^−2^ m and 1.9*10^−2^ m of inner diameter. The air linear speeds (m/s) applied during the tests are shown in Table 1:

**Table 1:** Air linear speeds (m/s) of gas flow applied during the study.

	PVC tubes inner diameter (m)	
Flow rate (m^3^/h)	1.9*10^−2^	4.5*10^−2^
**1.0–1.25**	1.0–1.2	0.2
**2.0–2.5**	2–2.2	0.4
**4.0–4.25**	3.9–4.2	0.7

### Agar diffusion inhibitory tests

Static microbial tests have been conducted using the agar disc diffusion method, with *Bacillus atrophaeus* DSM 675 as the selected bacteria strain. 150μL of this strain were put in 2mL of a solution of modified chopped meat growth medium (American Type Culture Collection medium 1490). The resulting suspension was incubated under continuous stirring at 303 K and 183 rpm for 4 hours. After incubation, a suspension containing approximately 4*10^5^ cells/mL has been prepared in 9g/L NaCl solution (Sigma-Aldrich).

5 mL of the diluted bacterial suspension was used to seed Tryptic Soy Agar (TSA) Petri dishes. After three minutes, the supernatant was taken out. The tested samples were then placed on the Petri dishes (7 beads per Petri, two Petri dishes per tested sample). The Petri dishes were incubated for 22 hours at 304 K. After incubation, the mean inhibition radii were measured using six radius inhibition measures.

### Test protocol to assess the germicidal efficiency under dynamic conditions

To follow the evolution of the concentration of microorganisms in the pilot device, the BioTrak 9510-BD was used. Equipped with a laser diffraction detector, this device counts all the particles occurring in the gas phase in the range of diameters from 0.5 to 25 μm (50% detection at 0.5 μm; 100% for particles >0.75 μm [23]) thanks to laser diffraction [24]. For the detection of viable particles from 1 to 25 μm, the BioTrak 9510-BD uses laser-induced fluorescence.

Two protocols were used; each one has been applied for different air flow rates.The first protocol was employed when using the polyvinyl chloride (PVC) sections having 4.5*10^−2^ m of inner diameter. During this test series 13 samples were collected by the Biotrak 9510-BD (volume of the sample: 14.3*10^−3^ m^3^, time of sampling: 30 s) with a 570 s break before each sampling operation. The whole procedure duration was 2 hours and 30 seconds (7230 s). This first protocol is not suitable for higher linear speeds of the gaseous phase as the decrease of the number of viable particles becomes so fast that it cannot be analyzed with the 2 hours and 30 seconds long protocol. The second protocol was used with the PVC sections having 1.9*10^−2^ m of inner diameter: 10 samples were collected by the Biotrak 9510-BD (volume of the sample: 14.3*10^−3^ m^3^, time of sampling: 30 seconds) with a 160 s break before each sampling operation. The whole procedure duration was 41 minutes (2460 s). For each of the six velocities applied, the tests were repeated three times to make sure the differences in the results are significant ones. The experiments have also been randomized.

## Results

### Scanning electron microscopy (SEM)

**Fig 3:**
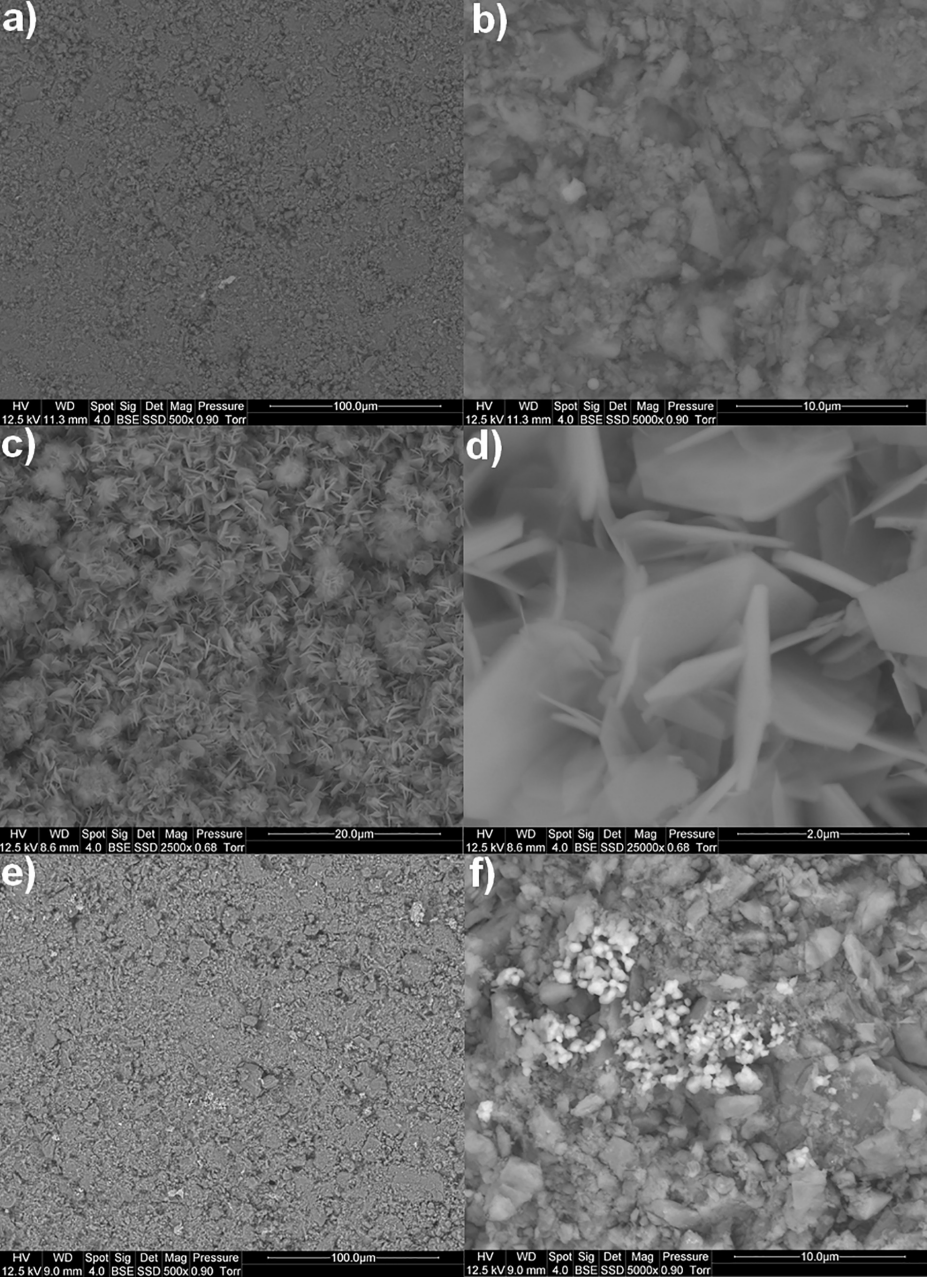
BSE SEM pictures of the tested materials. a, b) the γ-Al_2_O_3_ beads; c, d) the ZnO/γ-Al_2_O_3_ beads and e, f) the MnO_2_/AlPO_4_/γ-Al_2_O_3_ beads.

Scanning electron microscopy (SEM) analyses carried out using back-scattered electrons (BSE) confirmed the presence of a zinc oxide ZnO coating. As shown in Fig 3C, the coating is macroscopically homogenous. It is constituted with microstructures shaped like “desert roses”. Small hexagonal nanosheets are intercropped in these assembled microstructured elements. The sides of the hexagons measure between 0.7 and 15 μm and their thicknesses are about 50–160 nm. It does not look like non-coated γ-Al_2_O_3_ beads which show a “glob”-like morphology (Fig 3A & 3B). There are no angular shapes at its surface; the globs measure, in average, several micrometers. Fig 3F shows that in the case of MnO_2_/AlPO_4_/γ-Al_2_O_3_ beads there are smaller aggregates, brighter in BSE than for the beads of γ-Al_2_O_3_. These aggregates are the MnO_2_ globs. Their diameters are about 1μm or fewer.

### Energy-dispersive X-ray spectroscopy (EDX)

**Fig 4:**
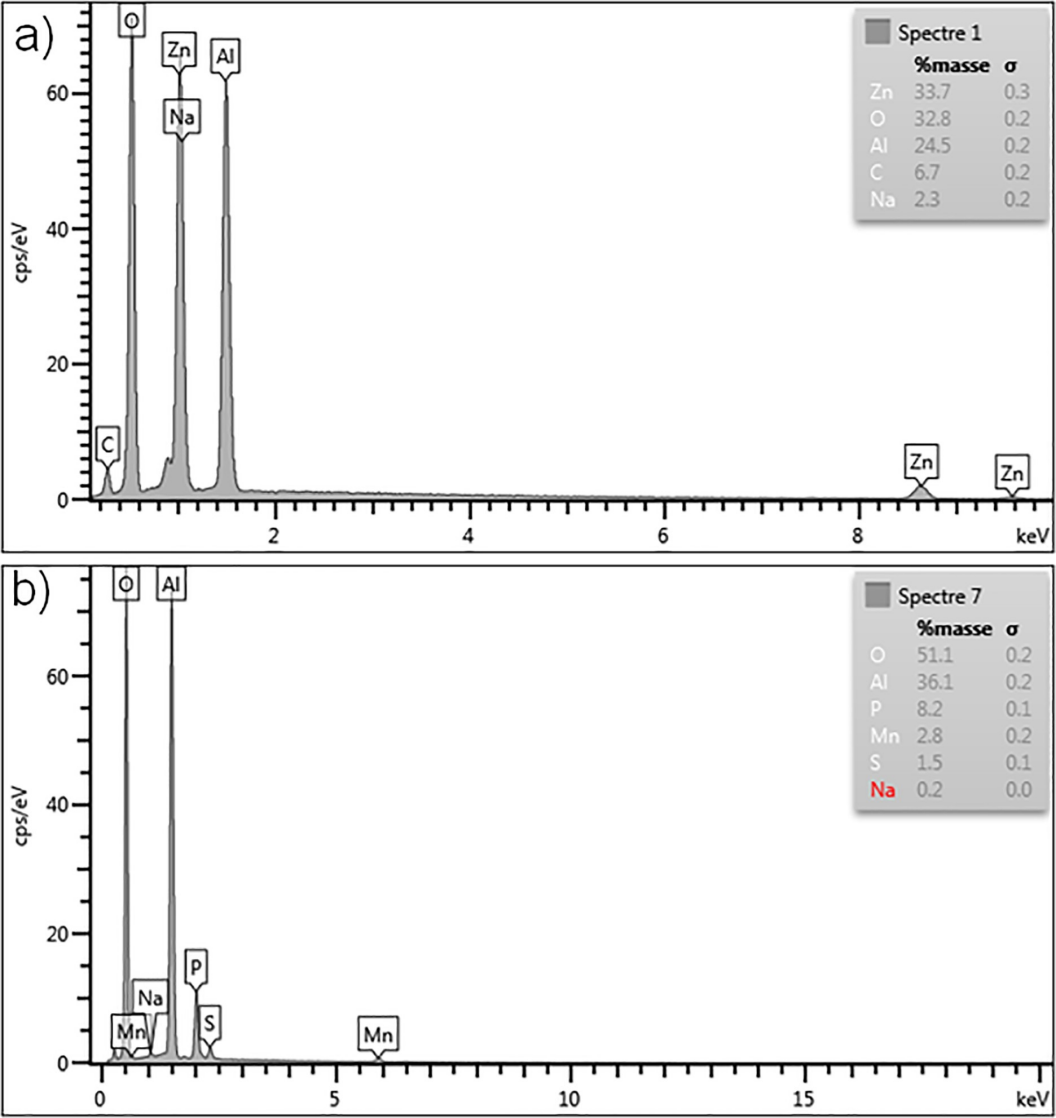
EDX analyses of the composite materials. a) ZnO/γ-Al_2_O_3_ beads and b) MnO_2_/AlPO_4_/γ-Al_2_O_3_ beads.

Together with the SEM analyses, an energy-dispersive X-ray spectroscopy (EDX) analysis was also carried out. Fig 4A shows the results obtained for the ZnO/γ-Al_2_O_3_ beads. The beads are in average made of 41.9 wt% of ZnO and 46.3 wt% of Al_2_O_3_.

Using the same method, the composition of the MnO_2_/AlPO_4_/γ-Al_2_O_3_ beads was determined (Fig 4B). In average, 4.5 wt% of manganese dioxide MnO_2,_ 32.3 wt% of AlPO_4_ and 54.8 wt% of Al_2_O_3_ were found on the surface of the beads.

### Data resulting from the surface area determination

The specific surfaces of all tested samples were determined by means of Brunauer–Emmett–Teller (BET) adsorption method using the Tristar II PLUS (Micrometrics). The results are displayed in Table 2. An important loss of specific surface area in MnO_2_-containing sample was noticed and attributed to the partial destruction of the support microporosity during the acid etching with H_3_PO_4_. The presence of the ZnO coating could also cause a partial pore closure but the developed surface of the ZnO microstructures could compensate partially this loss.

**Table 2:** Specific surface area of the tested materials.

Sample	Surface area (m^2^/g)
γ-Al_2_O_3_ beads	230
ZnO/γ-Al_2_O_3_ beads	198
MnO_2_/AlPO_4_/γ-Al_2_O_3_ beads	60

### Agar diffusion inhibitory tests

**Fig 5:**
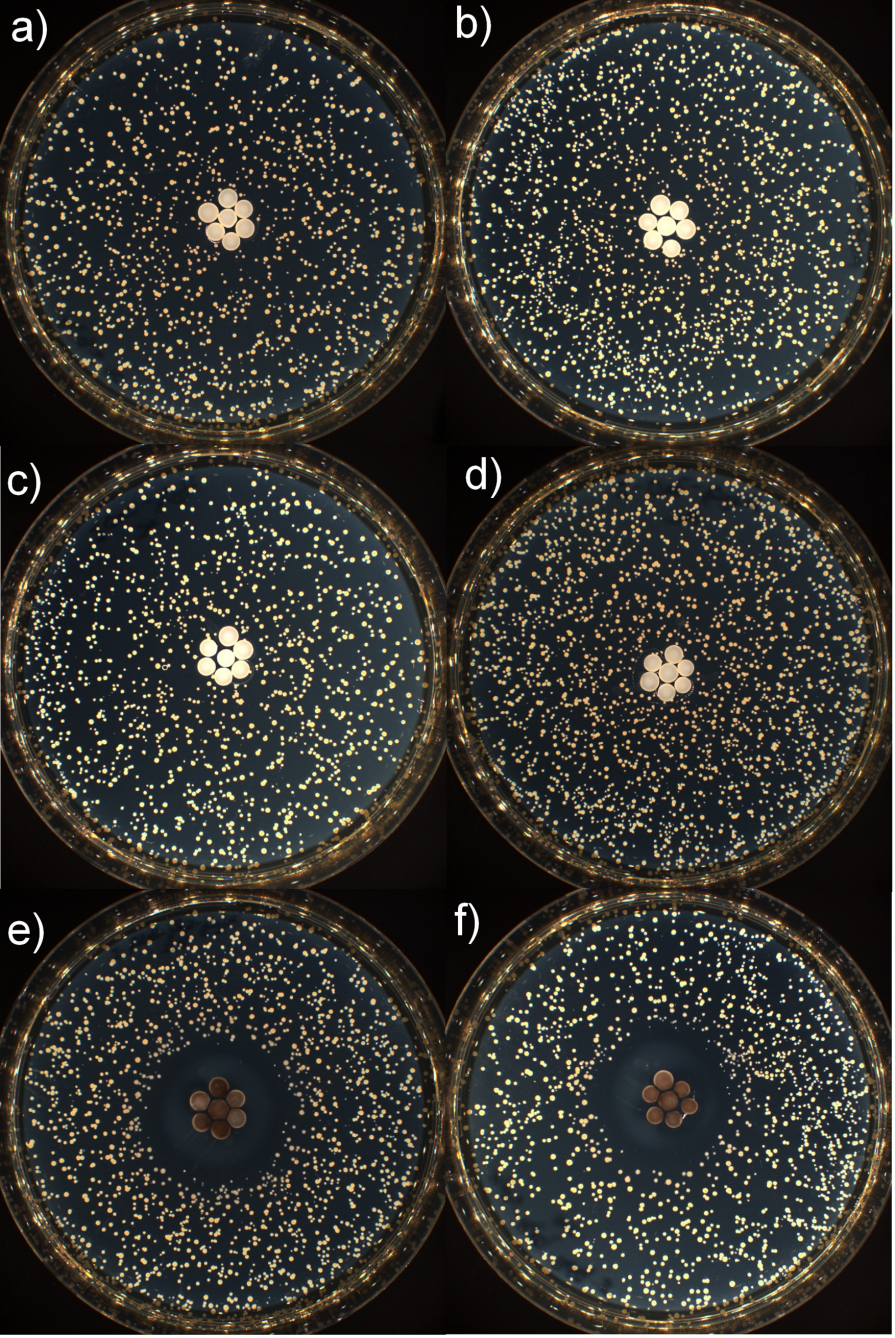
Agar diffusion inhibitory tests results. Incubated Petri dishes with *B*. *atrophaeus* and a, b) γ-Al_2_O_3_ beads, c, d) ZnO/γ-Al_2_O_3_ beads and e, f) MnO_2/_AlPO_4_/γ-Al_2_O_3_ beads.

The images of the incubated Petri dishes are displayed in Fig 5 and the mean inhibition radii are displayed in Table 3.

**Table 3:** Inhibition radii obtained by agar diffusion inhibitory tests.

	γ-Al_2_O_3_ beads	ZnO/γ-Al_2_O_3_ beads	MnO_2_/AlPO_4_/γ-Al_2_O_3_ beads
Inhibition radius of the first plate	0 mm	0 mm	9 mm
Inhibition radius of the second plate	0 mm	0 mm	9 mm
Mean inhibition radius	0 mm	0 mm	9 mm

As it can be seen on Fig 5, the only sample having an inhibition zone is the MnO_2_/AlPO_4_/γ-Al_2_O_3_ composite material. This implies that neither the non-modified γ-Al_2_O_3_ beads nor the ZnO/γ-Al_2_O_3_ beads manifest germicidal ability in the selected test conditions.

### Germicidal efficiency dynamic tests

#### Influence of the presence of a sample in the pilot device

**Fig 6:**
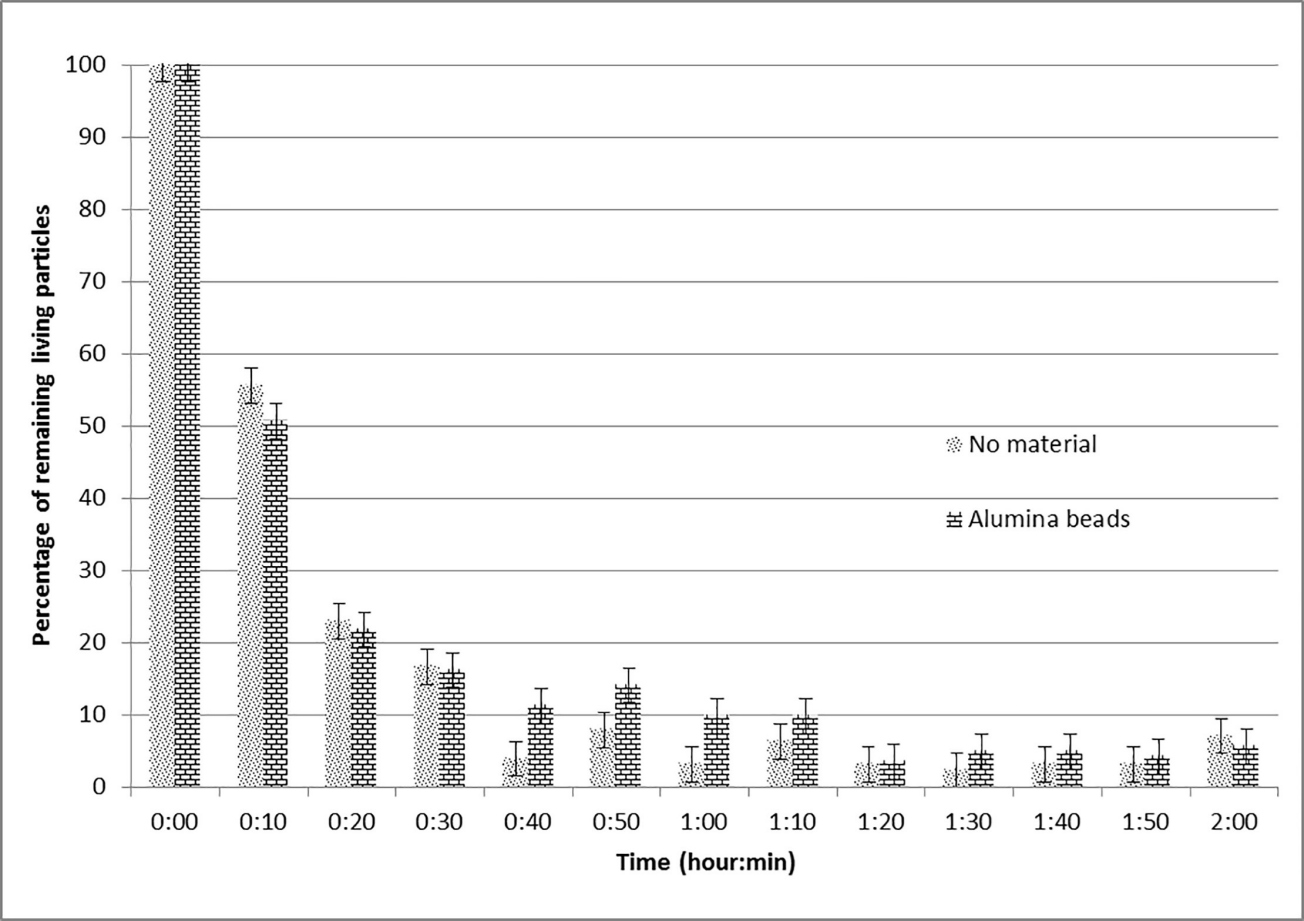
Evolution of the remaining percentage of microorganisms without and with alumina beads. Air linear speed: 0.2 m/s.

The data resulting from experiments without and with non-modified γ-Al_2_O_3_ beads are shown in Fig 6. The percentage of microorganisms remaining alive during the process of air circulation, both using an empty circuit and the circuit supplied with the sample under testing, has been followed-up in real time. This approach implies that the first measure always resulted in 100%. Therefore, it was decided to remove this point from the graphs for the other experimental conditions tested. It can be concluded that there is no substantial influence of the presence of a neutral non-modified solid material on the concentration levels of microorganisms in the circuit.

#### Tests with the two hours long protocol (first protocol)

**Fig 7:**
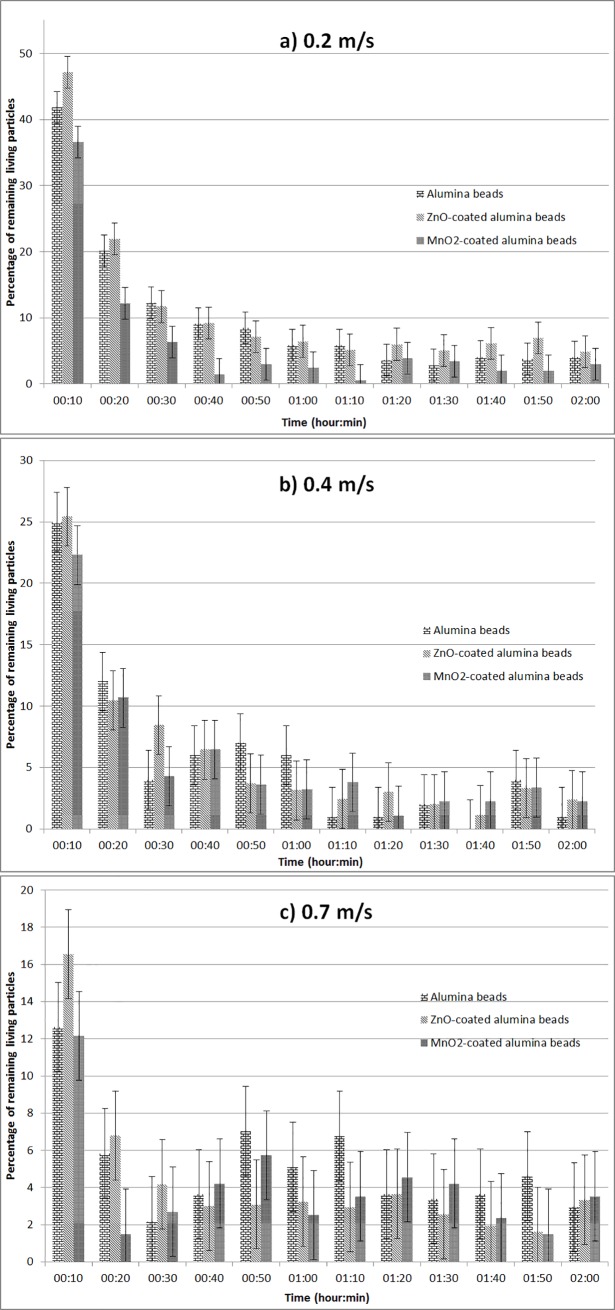
Test of the germicidal efficiency of materials in dynamic operating conditions. Air linear velocities: a) 0.2 m/s, b) 0.4 m/s, and c) 0.7m/s.

Non-modified γ-Al_2_O_3_ beads, ZnO/γ-Al_2_O_3_ beads and MnO_2_/AlPO_4_/γ-Al_2_O_3_ beads were tested using the first protocol at 0.2, 0.4 and 0.7 m/s linear air speed. The results are presented in Fig 7.

As shown in Fig 7A, at 0.2 m/s, the MnO_2_/AlPO_4_/γ-Al_2_O_3_ beads manifest higher performance than the two other materials. Indeed, it needs 40 minutes to clean out 95% of the initial quantity of bioaerosols. It is twice as efficient as with the non-coated alumina beads which need 1h20 to achieve the same result. In 30 minutes with a 0.2 m/s air linear speed the air goes through the system twice. In one hour, respectively, it goes through the system four times. When the ZnO/γ-Al_2_O_3_ beads are applied, during the first hour the microbial concentration decrease follows practically the same way as non-coated alumina beads. This fact may be due to a partial discharge of the microorganisms remaining alive from the surface of the sample. During the second part of the experiment, probably from the third entrance of the treated air into the circuit, an important decrease of the number of airborne microorganisms was observed.

As illustrated by Fig 7B, at 0.4 m/s the microbial decrease occurs faster. The MnO_2_/AlPO_4_/γ-Al_2_O_3_ beads are still the most efficient as they eliminate 95% of the initial microbial population in 30 minutes with a slight discharge at 40 minutes approximately. With the γ-Al_2_O_3_ beads, the concentration of microorganisms drops to 5% of its initial value in 30 minutes too but the discharge phenomenon which happens thereafter is more important. ZnO/γ-Al_2_O_3_ beads do not manifest a discharge phenomenon and eliminate 95% of the initial population of microorganisms in 50 minutes, which is still less efficient than in the case of MnO_2_/AlPO_4_/γ-Al_2_O_3_ beads.

At 0.7 m/s, the ZnO/γ-Al_2_O_3_ beads become the only material without a discharge phenomenon which inhibits 95% of the airborne microorganisms in 30 minutes. The MnO_2_/AlPO_4_/γ-Al_2_O_3_ beads make the microbial population decrease down to 5% of its original value in just 20 minutes. However, a discharge then occurs and stability under 5% of the initial population is reached at 1 hour. The γ-Al_2_O_3_ beads perform poorly as they reach the 5% threshold for good after 1h20.

The results obtained with the γ-Al_2_O_3_/AlPO_4_/MnO_2_ beads were already foreseen before the series of dynamic tests: in the case of photocatalytic air sanitation which also involves chemical surface reactions, the reactive oxygen species (ROS) generators require to be exposed to the treated media for relatively long periods of time. For instance, at the 0.2 m/s air linear velocity, the contact time between the tested ROS-generating material (MnO_2_/AlPO_4_/γ-Al_2_O_3_ beads) and the indoor air was 0.2 s. A contact time equal or superior to some tenths of seconds is absolutely necessary both in the case of photocatalytic and dark-operating oxidative antimicrobial air conditioning because the lysis of the bacteria and fungi is dominantly carried out in both cases by the adsorbed hydroxyl radicals HO∙_ads_.

#### Tests with the forty-one minutes long protocol (second protocol)

**Fig 8:**
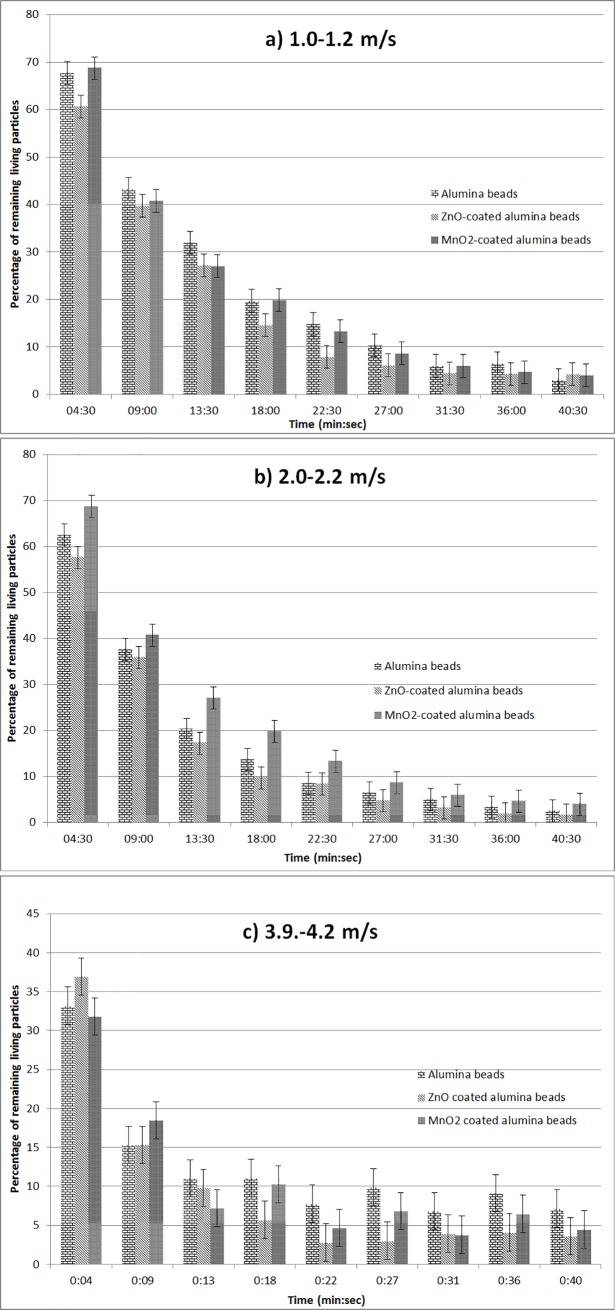
Test of the germicidal efficiency of materials in dynamic operating conditions. Air linear velocities: a) 1.0–1.2 m/s, b) 2.0–2.2 m/s, and c) 3.9–4.2 m/s.

The non-coated alumina beads, the ZnO/γ-Al_2_O_3_ beads and the MnO_2_/AlPO_4_/γ-Al_2_O_3_ beads were tested with the second protocol, at 1.0–1.2, 2.0–2.2 and 3.9–4.2 m/s linear air speed. The obtained results are shown in Fig 8.

At 1.0–1.2 m/s, the MnO_2_/AlPO_4_/γ-Al_2_O_3_ beads and the non-modified γ-Al_2_O_3_ beads have similar decrease paths, reaching 95% of viable cells removal at the end of the experiment, whereas the ZnO/γ-Al_2_O_3_ beads need 31 minutes and 30 seconds to do the same. The same pattern can be observed when applying a 2.0–2.2 m/s air flow speed. Indeed, when applying a 2.0–2.2 m/s air linear speed, the ZnO/γ-Al_2_O_3_ beads eliminate 95% of the initial quantity of bioaerosols in 26 minutes. The two other tested materials need 31 minutes and 30 seconds to do the same. When doubling the air linear speed and reaching 3.9 to 4.2 m/s, the ZnO/γ-Al_2_O_3_ beads keep being the most efficient material (95% viable particles removal in 22.5 minutes) and do not manifest any reportable discharge behavior. With the MnO_2_/AlPO_4_/γ-Al_2_O_3_ beads, the decrease of the microbial population is also 22.5 minutes long but the microbial concentration remains between 4 and 7% of its initial value. The non-modified γ-Al_2_O_3_ beads, however, do not achieve 95% of removal efficiency in these conditions.

## Discussion

The obtained results testify major differences in the behavior of the two types of developed dark-operating germicidal materials (DOGM).

The germicidal activity of the MnO_2_-based interactive composites (ROS-DOGM, ROS for reactive oxygen species, or 1^st^ dark-operating germicidal material type) can be attributed to a significant oxidative capacity of manganese dioxide.

In the chosen conditions of synthesis for the MnO_2_/AlPO_4_/γ-Al_2_O_3_ samples (calcination at 823 K in air for 4 hours), manganese dioxide crystallizes preferably in β-MnO_2_, a crystallographic analogue of a natural mineral pyrolusite. The β-MnO_2_ manifests the properties of *n*-semiconductor [25]. Its structure unit is a MnO_6_^8-^ octahedron (*O*_*h*_ symmetry) [26, 27].

As to the oxides of transition metals, due to a strong splitting effect in crystal field of electronegative ligands (oxygen anions), the *d*-electron states of oxide-forming elements occur both in conduction band edges and in valence band edges. It is also the case of manganese dioxide. The crystal field of the six O^2-^ anions splits the degeneracy of *3d* electron orbitals of manganese in two states–the lower energy *t*_*2g*_ triplets (the top of valence band positioned above the energetic levels of the oxygen anions) and the higher energy *e*_*g*_ doublets (the bottom of the conduction band). The *t*_*2g*_ triplets orbitals are totally occupied (octahedral manganese cations have *d*^*3*^ high-spin configuration), while the *e*_*g*_ doublet orbitals stay empty [28].

The main peculiarity of the *3d* orbitals splitting of the Mn-cation in manganese dioxide is the following: in an octahedral crystal field of the oxygen ligands the *t*_*2g*_ spin-down states are drifted very closely to the *e*_*g*_ spin-up states forming thus an extremely narrow bandgap announced to be as small as 0.25–0.28 eV (for β-MnO_2_) [25, 29, 30, 31]. As the result, a transition of an electron through the bandgap of β-MnO_2_ becomes relatively easy.

Indeed, the bandgap width equal to 0.25 eV is equivalent to a photon wavelength of 4959 nm placed deeply in the infrared part of the spectrum. The relationship between the temperature of a radiation-emitting body and the wavelength of its most intensive emittance is established by Wien’s law. Applying this law, it can be shown that the mentioned irradiance wavelength (4959 nm) relates to the thermal irradiation level at 583K, a temperature much higher than the ambient temperature used during this study.

Nevertheless, MnO_2_-based free and supported catalysts are successfully applied for the indoor air oxidative treatment even at room temperature [32]. Moreover, the experimental results obtained during the agar diffusion inhibitory tests (Fig 5 and Table 3) and the dynamic tests (Figs 7 and 8) are consistent with an oxidative action of the MnO_2_AlPO_4_/γ-Al_2_O_3_ material. This brings the question on the mechanism involved because, according to Wien’s law, the transition of electrons through the bandgap of manganese dioxide is impossible at room temperature.

A probable explanation of the observed effect may be the following: the MnO_2_AlPO_4_/γ-Al_2_O_3_ composite, containing an active component (β-MnO_2_) with an extremely narrow bandgap, is able to generate at its surface electron holes because of a fairly high probability of the tunneling effect.

In fact, the transparency for an electron of a 2D (flat) rectangular potential barrier–in particular, of a bandgap–in the simplest case can be evaluated as follows:

$$D=D_{0}*e^{\frac{-2d}{\hbar }\sqrt{2m_{e}\left(U_{0}-E\right)}},$$ (1)

where *D*–coefficient of transparency to be determined (0 < *D* ≤ 1); *D*_*0*_ –coefficient of transparency without any energetic barrier to overcome (*D*_*0*_ = 1); *m*_*e*_−masse of electron (9.11⋅10^−31^ *kg*); *ħ*–reduced Planck constant (*ħ* = *h* / 2π = 1.05⋅10^−34^ *J*⋅*s*); (*U*_*0*_ *–E*)–difference between the barrier’s height and the electron’s energy, *eV*; *d–*thickness of the barrier, *nm*.

The following variables can be used as basic data:

- (*U*_*0*_ *–E*) = 0.25 eV, where *U*_*0*_ = 0.25 eV (the bandgap energetic barrier),
- *E* = 0 eV (the worst virtual case when the electron’s own energy is not taken into consideration),
- *d* = 2.21 Å or 0.221 nm (the length of Mn_CB_–O_VB_ bond in MnO_2_ [30], where the indications “CB”, “VB” signify the ion’s position in the conduction band and in the valence band, respectively.

By substituting the mentioned data in the Eq (1) one can find the *D* value reaching 0.32 or 32%. Without any external energetic assistance one third of the electrons occurring in the valence band of manganese dioxide could be therefore transferred into the conduction band by means of tunneling.

Certain electrophysical properties of manganese dioxide are set out in Table 4. The concentrations of free charge carriers in manganese dioxide are relatively elevated, and so is its electrical resistivity. This is probably caused by low mobility of free electrons in a given *Oh*-architectured crystalline structure constructed with MnO_6_^8-^ units. The electrical conductivity of manganese dioxide is thus considered to be essentially determined namely by the electron mobility.

**Table 4:** Certain electrophysical properties of the manganese dioxide.

n°	Property	Character or value	Reference
1	Conductivity type	*n*-	[25]
2	Origins of the conducting properties	Tunneling effect	–
3	Electrical conductivity, *S*^∙^*cm*^*-1*^	From ≈ 10^−8^ to (3.2–12.7)^∙^10^−5^	[33, 34]
4	Electrical resistivity, *Ohm*^∙^*cm*	≈ 10^2^	[35]
5	Free charge carrier concentration, *cm*^*-3*^	(3.5–7.0)⋅10^18^	[34, 35]
6	Free charge carrier mobility, *cm*^*2*∙^*(V*^∙^*s)* ^*-1*^	**>** 10^**−**2^	[35]

A relatively high concentration of free charge carriers in manganese dioxide in its common non-excited state contributes to the reinforcement of the hypothesis that the valence band of this compound could be partially deprived of its electrons and hence be enriched in holes. Taking into account a high probability of the tunneling effect in β-MnO_2_ at room temperature, the existence of a significant number of holes at the surface of β-MnO_2_ in standard conditions seems to be quite realistic.

However, an important hole content of pure β-manganese dioxide cannot fully explain its potentially high oxidizing ability. Indeed, it seems difficult to justify the presence at the surface of pure β-MnO_2_ of oxidative reactive oxygen species (ROS) such as hydroxyl radicals HO∙. To successfully generate hydroxyl radicals on an oxide surface according to a simplified reaction scheme comprising the elementary reactions (2–4) [36], the hole redox potential must be sufficiently high in order to proceed the reaction (4) as it takes place in photocatalytic and electrocatalytic processes carried out using oxide active materials:

$$\equiv M^{n+}–O_{surf}^{2-}\to \equiv M^{n+}–O_{surf\;h+(VB)}^{-}+1\;e_{\left(CB\right)}^{-},$$ (2)

$$\equiv M^{n+}–O_{surf\;h+(VB)}^{-}+{HO}^{-}\to \equiv M^{n+}–O_{surf\;h+(VB)}^{-}–{HO}_{ads}^{-},$$ (3)

$$\equiv M^{n+}–O_{surf\;h+(VB)}^{-}–{HO}_{ads}^{-}\to \equiv M^{n+}–O_{surf}^{2-}–{HO}_{ads}^{•},$$ (4)

where M^n+^ signifies a cation of metal, O^2-^_surf_−an oxygen anion placed at the surface and having the 1s^2^2s^2^2p^6^ electronic configuration, O^-^_surf h+(VB)_−a charge deficient oxygen anion placed at the surface and having 1s^2^2s^2^2p^5^ electronic configuration, e^-^_(CB)_−an electron transferred into conduction band, h^+^_(VB)_−an electronic hole remaining in the valence band.

In the case of β-MnO_2_ the hole redox potential corresponds to the energetic distance between the filled higher occupied (HOMO) and the lower unoccupied (LUMO) molecular orbitals which is equal to the band gap value (0.25–0.28 eV). This energetic level has to be considered insufficient to favor the anodic partial oxidation of water according to the reaction (4).

A high oxidation ability of the developed MnO_2_-containing composite materials cannot be either adequately described in terms of presence in the samples of other crystalline modifications of the manganese dioxide.

The birnessite δ-MnO_2_, a graphite-like layered crystalline modification of the manganese dioxide, might be probably designated as a “good” candidate having the confirmed band gap values between 1.8 and 2.5 eV [37–39] and known as a promising catalyst for water-splitting processes carried out under sunlight irradiation. The photocatalytic behavior of δ-MnO_2_ confirms thus its ability to play a role of hydroxyl radical generator.

However, the tested MnO_2_-containing composite samples function reliably in total obscurity, whereas for the activation of birnessite a light irradiation is required. Besides of it, the applied MnO_2_-containing composites were developed in the experimental conditions (final treatment in air at 823 K for 4 hours) that promote the formation of the pyrolusite-type phase β-MnO_2_ and not of the birnessite which is mainly formed in moderate thermal conditions in humid media (for example, in sea water) [38]. Nevertheless, some rare references communicate that a layer-structured crystalline modification of the manganese dioxide may be obtained in free (powdered) form by means of thermal decomposition of KMnO_4_ when heating in air from 298 K to 773 K using the heating rate of 5 K / min [40].

Therefore, an explanation is needed for the important oxidation capacity of the tested MnO_2_-containing composite materials. To elucidate this, a probable interactivity resulting from continuous donor-acceptor interactions between the manganese dioxide (Lewis base, donor component) and the phosphate of aluminium / activated alumina (Lewis acid, acceptor component) should be taken into account.

Due to the applied method of synthesis, all components of the developed composite material remain chemically bound and form thus new donor-acceptor covalent clusters with [H^∙∙∙∙∙^O_VB(D)_]_ext_–[Mn_CB(D)_–O_VB(A)_–Al]_int_ and [H^∙∙∙∙∙^O_VB(D)_]_ext_–[Mn_CB(D)_–O_VB(A)_–P]_int_ cross-linking bonds at the surface. The external spheres of these clusters are constituted with Bronsted acid sites. The subscript “VB(D)” indicates the belonging of all external oxygen anions to the valence band of the donor component. In turn, the internal cluster spheres have mixed electronic structures: the manganese cations constituting the donor’s conduction band (“CB(D)”) are chemically linked to the oxygen anions playing the role of bridging atoms and creating the valence band of the acceptor support (“VB(A)”).

Inside of the mentioned donor-acceptor clusters a part of the electrons placed in the manganese dioxide valence band (VB_MnO2_) may be considered as free charge carriers (FCC): these electrons are able to move freely from the VB_MnO2_ to its conduction band (CB_MnO2_). According to the evaluation results obtained using the Eq (1), even for pure β-MnO_2_ the FCC electron part corresponds approximately to one third of the total electron number. As to the clusters [H^∙∙∙∙∙^O_VB(D)_]_ext_–[Mn_CB(D)_–O_VB(A)_–Al]_int_ and [H^∙∙∙∙∙^O_VB(D)_]_ext_–[Mn_CB(D)_–O_VB(A)_–P]_int_, the FCC part can be noticeably greater due to intensive electron pumping on the side of acceptor supports.

**Fig 9:**
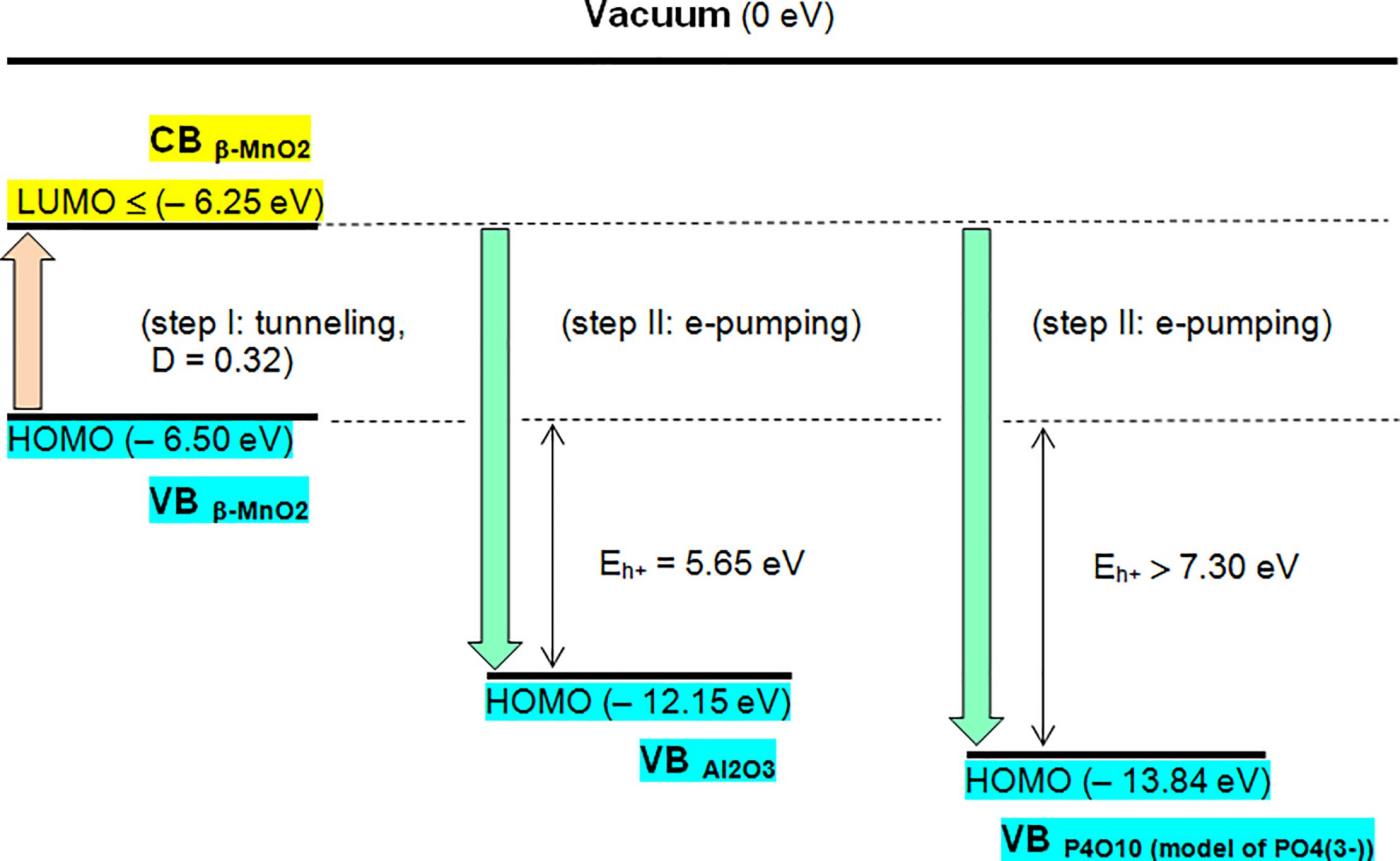
Functional energetic pattern of the MnO_2_/AlPO_4_/γ-Al_2_O_3_ composites.

In the case under consideration the FCC occurring in the CB_MnO2_ and being located, at the same time, within the free movement spaces (FMS) which are covalent donor-acceptor clusters, have to be energetically best positioned. Inside the FMS, the FCC cannot return to the VB_MnO2_ because the manganese dioxide HOMO (Highest Occupied Molecular Orbital) edge is situated at a considerably higher energy level than the ones of Al_2_O_3_ and AlPO_4_ (Table 5). The directions of electron transfer inside pure β-MnO_2_ and the donor-acceptor interactive composites MnO_2_/AlPO_4_/γ-Al_2_O_3_ are schematically presented in Fig 9.

**Table 5:** Energetic characteristics of donor and acceptor components of the MnO_2_/AlPO_4_/γ-Al_2_O_3_ composites.

Donor oxide component (D) and its acceptor support (A)	β-MnO_2_ (D)	Al_2_O_3_ (A)	P_4_O_10_ (A) (model of PO_4_^3-^)
Absolute HOMO position corresponding to the oxide’s work function, eV	(– 6.50) [29]	(– 12.15) [41]	(– 13.84) [41]
Relative HOMO position ΔE_HOMO_ = HOMO_MnO2_ –HOMO_A_, eV	–	5.65	**>** 7.30
Estimative energy of electron holes h^+^ occurring in the donor’s valence band VB_MnO2_ for pure β-MnO_2_ and for its donor-acceptor composites (separately for MnO_2_/Al_2_O_3_ and for MnO_2_/AlPO_4_), eV	0.25–0.28 E_h+ MnO2_ = LUMO_MnO2_ –HOMO_MnO2_ [25, 29, 30, 31]	5.65 E_h+ MnO2/Al2O3_ = HOMO_MnO2_ –HOMO_Al2O3_	**>** 7.30 E_h+ MnO2/AlPO4_ = HOMO_MnO2_ –HOMO_P2O10 (model)_

LUMO—Lowest Unoccupied Molecular Orbitals.

According to the functional energetic pattern shown in Fig 9 the electron transfer inside of the MnO_2_/AlPO_4_/γ-Al_2_O_3_ donor-acceptor interactive composites is carried out in two consecutive steps. Firstly, a part of electrons leaves from the manganese dioxide valence band (step I) due to the tunneling effect. Secondly, the appeared FCC are transferred within the internal spheres of the created covalent clusters [H^∙∙∙∙∙^O_VB(D)_]_ext_–[Mn_CB(D)_–O_VB(A)_–Al]_int_ and [H^∙∙∙∙∙^O_VB(D)_]_ext_–[Mn_CB(D)_–O_VB(A)_–P]_int_ from the CB_MnO2_ towards the valence bands of the acceptor components: their HOMO are positioned at lower energies than the HOMO of β-MnO_2_. The electron holes h^+^ created in the VB_MnO2_ by the transfer process mentioned above are significantly more powerful than the holes of pure β-MnO_2_ (Table 5, Fig 9). Their oxidative potentials largely overcome the ones of the holes which can be created by an absolute majority of the used photo- and electrocatalysts proceeding at their surfaces the elementary reactions (2–4). The hole oxidative potentials exactly correspond to the band-gap widths of applied active materials and, in most cases, lay in the range from 2.5 to 3.5 eV.

Elevated hole oxidative potentials of the developed MnO_2_-based interactive composites (reactive oxygen species-DOGM or 1^st^ DOGM type) ensure therefore their ability to proceed with the elementary reactions (2–4) without any external excitation, only by means of continuous donor-acceptor interactions inside of the covalent clusters [H^∙∙∙∙∙^O_VB(D)_]_ext_–[Mn_CB(D)_–O_VB(A)_–Al]_int_ and [H^∙∙∙∙∙^O_VB(D)_]_ext_–[Mn_CB(D)_–O_VB(A)_–P]_int_. This conclusion is objectively proven by the results of inhibition radius tests (Fig 5C and 5D).

As to the results obtained during the tests carried out in dynamic conditions (Figs 7 and 8), it should be noted that these results remain in whole compliance with prior expectations.

For the MnO_2_/AlPO_4_/γ-Al_2_O_3_ composite samples the best germicidal performances were observed at moderated flow velocities: these operating conditions provide significant contact times. These contact times are imperatively required for pertinent commitment of adsorbed hydroxyl radicals into the sanitation process, in the same way as for photocatalysis. On the contrary, the ZnO/γ-Al_2_O_3_ composite samples function noticeably better at relatively high flow velocities, which is consistent with an abrasive behaviour that does not need important contact times.

At the same time, there is a little likelihood that the reactions (2–4) would be carried out at the surfaces of the ZnO/γ-Al_2_O_3_ composites (Mecha-DOGM or 2^nd^ dark-operating germicidal material type). Using the formula (1) on can evaluate a probability of the tunneling effect in ZnO: (*U*_*0*_ *–E*) = 3.73 eV, where *U*_*0*_ = 3.73 eV (the bandgap energetic barrier) [38], *d* = 1.98 Å or 0.198 nm (the length of Zn_CB_–O_VB_ bond in ZnO [42]), D ≈ 0.02 or only 2%. No diffusive oxidation stress was observed for bacteria strains during the agar diffusion inhibitory tests of the ZnO/γ-Al_2_O_3_ composite samples (Fig 5E and 5F). These composites hence perform their sanitation ability exclusively in dynamic flow conditions and act thus as mechanical cell destructors.

It seems important to mention that the geometrical characteristics of mechanically-obstructive elements placed at the surface of the developed ZnO/γ-Al_2_O_3_ composite material are perfectly in keeping with typical bacterial cell dimensions (Fig 3D), and that could explain the significant germicidal efficiency observed.

Taking into account the experimental results shown in Fig 8, an evaluation the efficiencies of the tested composites against airborne bacteria in real dynamic conditions is possible.

The estimative values of the composites germicidal efficiencies obtained regarding the number of air runs in the pilot device can be estimated using an exponential model for the microbial concentration decrease. The results for a 2.5 m^3^/h flow rate and 2.0–2.2 m/s air velocity, which correspond to a six minutes air run, are shown in Table 6.

**Table 6:** Estimative germicidal efficiencies of the ZnO/γ-Al_2_O_3_ and MnO_2_/AlPO_4_//γ-Al_2_O_3_ composites for a 2.0–2.2 m/s gas linear velocity.

Number of the air runs across the test device during the treatment procedure		1	2	3	4	5	6
Model application case		Antimicrobial door air curtain (single air run)	Air circulation system with integrated antimicrobial device (several air runs)				
Estimative germicidal efficiency (%)	ZnO/γ-Al_2_O_3_ beads	55	76	87	93	> 95	>95
	MnO_2_/AlPO_4_//γ-Al_2_O_3_ beads	45	67	80	88	82	95

It may be seen that, at relatively moderated gas linear velocities and with only single indoor air run through the ZnO/γ-Al_2_O_3_ granular layer, a considerable part of airborne bacteria can be removed from the gas medium and completely destroyed (no secondary discharge effect was observed in time). It is worthwhile to underline that the applied Mecha-DOGM had to operate under harsh dynamic conditions, at an extremely high volumetric flow velocity calculated as the quotient of the gas flow rate divided by the apparent volume of the used active material. This fact tends to prove a high productivity of the ZnO/γ-Al_2_O_3_ composite material when applied for dynamic antimicrobial conditioning of the indoor air.

As also follows from the data presented in Table 6, the Mecha-DOGM manifest the highest germicidal efficiencies in circulation treatment mode: several air runs are sufficient in order to guarantee very important germicidal efficiency levels.

## Conclusion

Static tests such as agar diffusion inhibitory test (ADT) are to this day the standard procedure to explore the germicidal potential of materials. However, as our study highlighted, the information these tests provided is simply not enough when searching for materials for the disinfection of indoor air. Indeed, some materials such as the ZnO/γ-Al_2_O_3_ beads seem to have a mechanical abrasive effect on microorganisms which cannot be observed in static conditions. With the dynamic tests, however, which approached life-like use of the materials (application of ambient air, constant air flow through the materials), the germicidal effect of this material was noticed. It is important to stress that the results of the tests were influenced by its operational conditions and especially by the air velocity. When employing an air velocity below 0.7 m/s, the MnO_2_/AlPO_4_/ γ-Al_2_O_3_ beads were the most efficient of the tested materials for the inactivation of the airborne microorganisms, which is consistent with the inhibition zone test results. Indeed, its structure allows the generation of hydroxyl radicals at room temperature without any energetic assistance thanks to tunneling effect and also to donor-acceptor interactions between MnO_2_ and AlPO_4_/γ-Al_2_O_3_. These hydroxyl radicals are a source of oxidative stress for microorganisms and are located on the surface of the beads, which explain that this material works best when using low speeds and thus high contact times. However, when applying higher velocities (between 0.7 and 4 m/s), the ZnO/γ-Al_2_O_3_ beads became the most effective material. This is concordant with an abrasive behavior. To conclude, testing the germicidal potential of materials in realistic conditions provided unique and key information. Therefore, it is to be hoped that the use of dynamic tests such as ours will be generalized in the near future for the research of materials for the disinfection of air.

## Supporting information

S1 Data Data set used in the present study for the germicidal efficiency dynamic tests. This file contains the data obtained for the experiments analyzed via the BioTrak 9510-BD. The data set has been sorted out to facilitate reading.
(XLSX)
